# Challenges of Caring for an Acute Population in a Disaster

**DOI:** 10.1093/geroni/igaa057.2609

**Published:** 2020-12-16

**Authors:** Lindsay Peterson, Kathryn Hyer, David Dosa, Joseph June, Debra Dobbs

**Affiliations:** 1 University of South Florida, Tampa, Florida, United States; 2 Brown University, Barrington, Rhode Island, United States; 3 School of Aging Studies, University of South Florida, Tampa, Florida, United States

## Abstract

The U.S. Gulf Coast hurricanes of 2004-08 led to research and policy reports highlighting the need for more emergency preparation among nursing homes (NH). In 2016, the federal government issued final rules requiring Medicaid and Medicare providers to develop comprehensive preparedness plans. The state of Florida previously imposed its own long-term care (LTC) preparedness requirements. Hurricane Irma tested the readiness of LTC facilities that care for disabled and vulnerable residents. This research examined the experiences of NHs (N=30) affected by the hurricane through qualitative interviews with administrative staff. Research team members analyzed the transcripts, identified codes, and met to reach consensus on themes. Three major themes emerged, 1) managing the unexpected, including last-minute evacuation orders, 2) caring for vulnerable residents amid the crisis, and 3) the struggle of maintaining staff. Results suggest LTC preparation has increased but long-standing problems continue, including conflicts with emergency management priorities.

